# Traditional Chinese Medication Qiliqiangxin Attenuates Diabetic Cardiomyopathy via Activating PPARγ

**DOI:** 10.3389/fcvm.2021.698056

**Published:** 2021-07-16

**Authors:** Xiaodong Wu, Ting Zhang, Ping Lyu, Mengli Chen, Gehui Ni, Huiling Cheng, Guie Xu, Xinli Li, Lijun Wang, Hongcai Shang

**Affiliations:** ^1^Department of Cardiology, The First Affiliated Hospital of Nanjing Medical University, Nanjing, China; ^2^Cardiac Regeneration and Ageing Lab, Shanghai Engineering Research Center of Organ Repair, School of Life Science, Institute of Cardiovascular Sciences, Shanghai University, Shanghai, China; ^3^Key Laboratory of Chinese Internal Medicine of Ministry of Education, Dongzhimen Hospital, Beijing University of Chinese Medicine, Beijing, China

**Keywords:** Qiliqiangxin, diabetic cardiomyopathy, apoptosis, fibrosis, PPARγ, PGC-1α

## Abstract

**Background:** Diabetic cardiomyopathy is the primary complication associated with diabetes mellitus and also is a major cause of death and disability. Limited pharmacological therapies are available for diabetic cardiomyopathy. Qiliqiangxin (QLQX), a Chinese medication, has been proven to be beneficial for heart failure patients. However, the role and the underlying protective mechanisms of QLQX in diabetic cardiomyopathy remain largely unexplored.

**Methods:** Primary neonatal rat cardiomyocytes (NRCMs) were treated with glucose (HG, 40 mM) to establish the hyperglycemia-induced apoptosis model *in vitro*. Streptozotocin (STZ, 50 mg/kg/day for 5 consecutive days) was intraperitoneally injected into mice to establish the diabetic cardiomyopathy model *in vivo*. Various analyses including qRT-PCR, western blot, immunofluorescence [terminal deoxynucleotidyl transferase-mediated dUTP nick-end labeling (TUNEL) staining] histology (hematoxylin–eosin and Masson's trichrome staining), and cardiac function (echocardiography) were performed in these mice. QLQX (0.5 μg/ml *in vitro* and 0.5 g/kg/day *in vivo*) was used in this study.

**Results:** QLQX attenuated hyperglycemia-induced cardiomyocyte apoptosis via activating peroxisome proliferation-activated receptor γ (PPARγ). *In vivo*, QLQX treatment protected mice against STZ-induced cardiac dysfunction and pathological remodeling.

**Conclusions:** QLQX attenuates diabetic cardiomyopathy via activating PPARγ.

## Introduction

Diabetes mellitus is a worldwide health problem with global incidence, and the number of diabetic cases were estimated to reach 550 million by 2030 (1–3). Diabetic patients always suffer from diverse complications, especially cardiovascular disorders which occupy 40–60% of total diabetic individuals, and cardiovascular mortality accounts for two-thirds of total mortality (3–6). Previous studies have reported that diabetic patients have a higher heart failure rate vs. non-diabetic patients, 2-fold in men and 5-fold in women (7–9).

Diabetic cardiomyopathy is characterized as ventricular dysfunction with metabolic disturbances, accompanied by apoptosis, hypertrophy, fibrosis, inflammation, and oxidative stress, and eventually leads to cardiac dysfunction and heart failure (10, 11). Diabetic cardiomyopathy was defined as a clinical condition independent of coronary atherosclerosis, hypertension in diabetic patients (7, 12). Cardiomyocyte apoptosis, the most frequently proposed mechanism resulting from hyperglycemia, causes severe loss of contractile tissue and subsequently initiates cardiac remodeling, including reactive oxygen species (ROS), inflammation, and structure disturbance (13–15). However, treatment strategies for preventing diabetic cardiomyopathy are still limited.

Qiliqiangxin (QLQX) is a Chinese medication that has been reported to be beneficial to heart failure patients with cardiovascular disorders (16). Previous studies have demonstrated that QLQX can attenuate cardiac remodeling after acute myocardial infarction and phenylephrine-induced cardiac hypertrophy (17, 18). However, whether QLQX could exert a beneficial effect on diabetic hearts remains unknown. Peroxisome proliferation-activated receptor γ (PPARγ), a transcriptional regulator, participates in the development of various diseases, including diabetes mellitus. PPARγ co-activator-1α (PGC-1α) can function as a co-activator of PPARγ, and PGC-1α signaling activation stimulated by PPARγ directly alters myocardial metabolism within the mitochondria (19). The agonist of PPARγ, thiazolidinediones (TZDs), can act as insulin-sensitizing drugs and has been implicated in diabetic therapy. PPARγ activation directly induces alteration in glucose and lipid utilization, takes charge of hyperglycemia, and develops further beneficial influence (10, 20). Stimulation of PPARγ inhibits cellular inflammatory response, suppresses myocardial fibrosis, and improves cardiac diastolic dysfunction in diabetic hearts (21, 22). Though PPARγ and PGC-1α play vital roles in QLQX-protecting cardiac function and attenuating cardiac remodeling, whether PPARγ activation contributes to QLQX-mediated diabetic heart benefit remains to be discovered.

Here, in our study, we found that QLQX has a cardioprotective role in hyperglycemia-induced cardiomyocyte apoptosis via activating PPARγ *in vitro*. *In vivo*, QLQX treatment protected mice from streptozotocin (STZ)-induced cardiac dysfunction.

## Materials and Methods

### Primary Neonatal Rat Ventricular Cardiomyocyte (NRCM) Isolation, Culture, and Treatment

Primary NRCMs were extracted from 1-day-old SD rats as previously described (18). After the cardiomyocyte extraction, cardiomyocytes were cultured in Dulbecco's modified Eagle's medium (DMEM) (Gibco, Pasadena, CA, USA) supplemented with 5% fetal bovine serum (FBS) (Biological Industries, Israel), 10% horse serum (HS) (Biological Industries, Israel), and 1% penicillin–streptomycin (Gibco, Pasadena, CA, USA).

To simulate a hyperglycemic environment, cardiomyocyte was exposed to serum-free DMEM containing 40 mM glucose [high glucose (HG)], while 5.5 mM glucose (control) was set as normal control. Forty-eight hours after HG exposure, cardiomyocyte was harvested.

To investigate if the Chinese medication QLQX can exert a protective effect on HG-treated cardiomyocyte, cardiomyocyte was pretreated with QLQX (0.5 μg/ml) for 48 h before HG exposure.

To further clarify the mechanism of how QLQX exerts its beneficial effect, cardiomyocytes were pretreated with PPARγ agonist (rosiglitazone, 1 μM) (Selleck Chemicals, Houston, TX, USA) or PPARγ inhibitor (T0070907, 1 μM) (Selleck Chemicals, Houston, TX, USA) along with QLQX treatment for 48 h, followed by HG exposure for another 48 h.

### Terminal Deoxynucleotidyl Transferase-Mediated dUTP Nick-End Labeling (TUNEL) Assay

Apoptosis in NRCMs and mouse heart sections was detected using a TUNEL apoptosis detection kit (Alexa Fluor 488) (Yeasen, Shanghai, China) according to the manufacturer's instruction. For *in vitro* analysis, NRCMs were incubated with α-actinin (Sigma-Aldrich, St Louis, MO, USA) overnight, followed by Cy3 AffiniPure Goat Anti-Mouse IgG (H+L) incubation for 2 h at room temperature. Then, NRCMs were subjected to TUNEL staining reagents, and nuclei were counterstained with DAPI. For detection in heart tissues, sections of heart tissues (5 μm) were subjected to TUNEL staining followed by DAPI staining. Fluorescence microscopy was used to capture TUNEL-positive nuclei in NRCMs or heart tissues. The percentage of TUNEL-positive cardiomyocytes was calculated to determine apoptosis in NRCMs or the hearts.

### Animals

This experiment was performed under the guidelines on the humane use and care of laboratory animals for biomedical research published by the National Institutes of Health (no. 85-23, revised 1996). All experimental protocols were approved by the ethical committees of the Nanjing Medical University (no. IACUC-1903016).

Male mice were obtained from Beijing Charles River Laboratory Animal Technology Corporation and housed in a room receiving a 12-h dark/light cycle and free access to food. Streptozotocin (STZ, Sigma-Aldrich, St Louis, MO, USA) was used to induce hyperglycemia and diabetic cardiomyopathy at a dosage of 50 mg/kg/day for 5 consecutive days via intraperitoneal injection. Before the STZ injection, basic blood glucose level of mice was examined from the tail vein using a glucometer (Roche, Mannheim, Germany). After 72 h of final STZ injection, the blood glucose level of mice was detected, and mice with a blood glucose level more than 11.1 mmol/L were thought to be diabetic. Subsequently, mice were treated with QLQX (0.5 g/kg/day) vs. saline via intragastric administration for 8 weeks. Blood glucose level was examined once every 2 weeks.

### Echocardiography

Mice were anesthetized with 2% isoflurane, and echocardiography was conducted using Vevo 2100 (Visual Sonics Inc., Toronto, Ontario, Canada). Cardiac systolic function was evaluated by measuring the parameters of the left ventricular ejection fraction (EF%) and left ventricular fractional shortening (FS%). Cardiac diastolic function was assessed by the ratio of early (E-wave) and late (A-wave) LV diastolic filling velocities (E/A ratio). All parameters examined by echocardiography are shown in Supplementary Table 1.

### Western Blot Analysis

Total proteins were extracted from NRCMs or heart tissues using RIPA buffer (KeyGen, Nanjing, China), and protein concentration was quantified by a BCA protein assay kit (Thermo Fisher Scientific, Waltham, MA, USA). Twenty micrograms of protein was loaded and separated on 12% SDS-PAGE gels and then transferred to a PVDF membrane. Subsequently, the membrane was blocked with 5% BSA and incubated with specific antibody as follows: PGC-1α (1:1,000, ProteinTech, Wuhan, China), PPARγ (1:1,000, ProteinTech, Wuhan, China), Bax (1:1,000, ProteinTech, Wuhan, China), Bcl-2 (1:1,000, ProteinTech, Wuhan, China), caspase 3 (1:1,000, ProteinTech, Wuhan, China), HO-1 (1:1,000, ProteinTech, Wuhan, China), TNFα (1:1,000, Servicebio, Wuhan, China), and IL-1β (1:1,000, ProteinTech, Wuhan, China). Membranes were incubated with HRP-linked secondary antibody, and the ChemiDoc XRS Plus luminescent image analyzer (Bio-Rad, Hercules, CA, USA) was used for detection. The GAPDH (1:1,000, ProteinTech, Wuhan, China) or tubulin (1:1,000, Servicebio, Wuhan, China) was used as a loading control. Uncropped scans of western blots are listed in Supplementary Figure 1.

### Quantitative Real-Time Polymerase Chain Reaction (qRT-PCR) Analysis

Total RNA from murine heart was extracted using the TRIzol reagent (TAKARA, Tokyo, Japan). To obtain cDNA, reverse transcription was subsequently performed using an iScript™ cDNA synthesis kit (Bio-Rad, Hercules, CA, USA). And the ABI-7900 real-time PCR system (Applied Biosystems, CA, USA) was used to detect the relative mRNA level with SYBR Green Master Mix (Yeasen, Shanghai, China). 18S functions as an inner control to normalize the mRNA expression followed by the 2^−ΔΔCT^ method. Primers involved in study are exhibited in Supplementary Table 2.

### Histological Analysis

The heart sections were fixed with 4% paraformaldehyde for further evaluation. As previously described (23), hematoxylin–eosin (H&E) staining was conducted to assess cellular cross-sectional area, and Masson trichrome staining was performed to evaluate cardiac fibrosis. Images were captured by a Nikon Eclipse microscope with the NIS Elements software, and ImageJ software was used for further analysis.

### Statistical Analysis

All data were analyzed and presented as mean ± SD using GraphPad Prism 6.0 (La Jolla, CA, USA). For comparison between two groups, two-tailed unpaired Student's *t-*test was used. And for multiple-group comparisons, one-way ANOVA or two-way ANOVA analysis followed by Bonferroni's *post-hoc* test was performed. A *p* < 0.05 was considered statistically significant.

## Results

### HG Promotes NRCM Apoptosis and Downregulates PPARγ and PGC-1α

**Figure 1:**
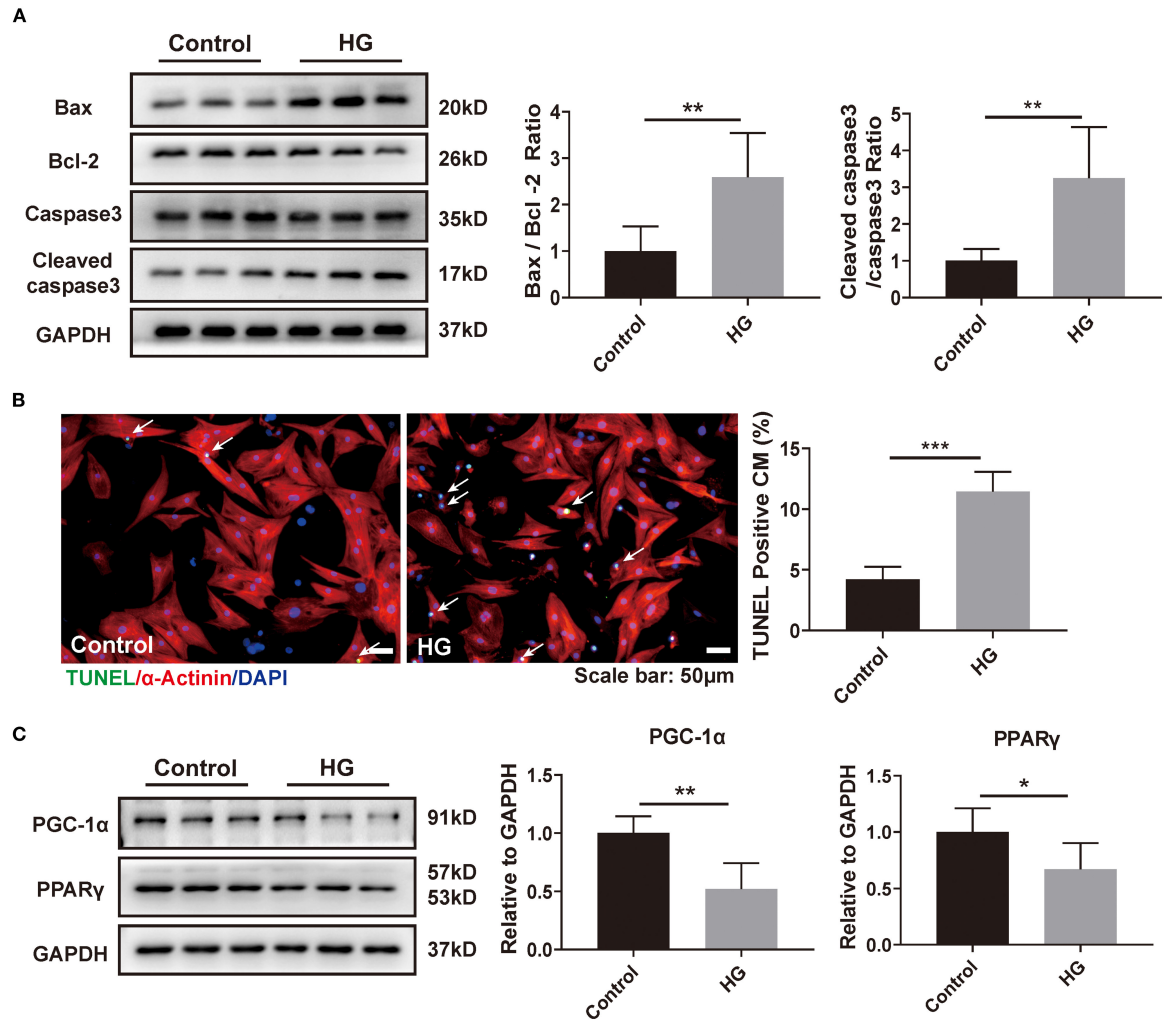
HG promotes NRCM apoptosis and downregulates PPARγ/PGC-1α. **(A)** Western blot analysis was used to evaluate cardiomyocyte apoptosis with or without HG exposure (*n* = 6). **(B)** TUNEL staining and quantitation analysis with or without HG treatment. Scale bar: 50 μm (*n* = 6). **(C)** Western blotting analysis of PPARγ and PGC-1α protein (*n* = 6). *p < 0.05; **p < 0.01; ***p < 0.001; vs. respective control.

Hyperglycemia, as a typical feature of diabetes mellitus, functions as the initial factor for diabetic complications (24). We cultured NRCMs with DMEM containing HG (40 mM) and found that, compared to that in the normal-glucose condition (control, 5.5 mM), cardiomyocyte apoptosis in the HG group was significantly increased as determined by western blot and TUNEL staining (Figures 1A,B). Besides, the expressions of PPARγ and its coactivator PGC-1α were obviously decreased in the HG group (Figure 1C), indicating that reduced PPARγ and PGC-1α might contribute to hyperglycemia-induced cardiomyocyte injury.

### QLQX Attenuates HG-Induced NRCM Apoptosis and Activates PPARγ/PGC-1α

**Figure 2:**
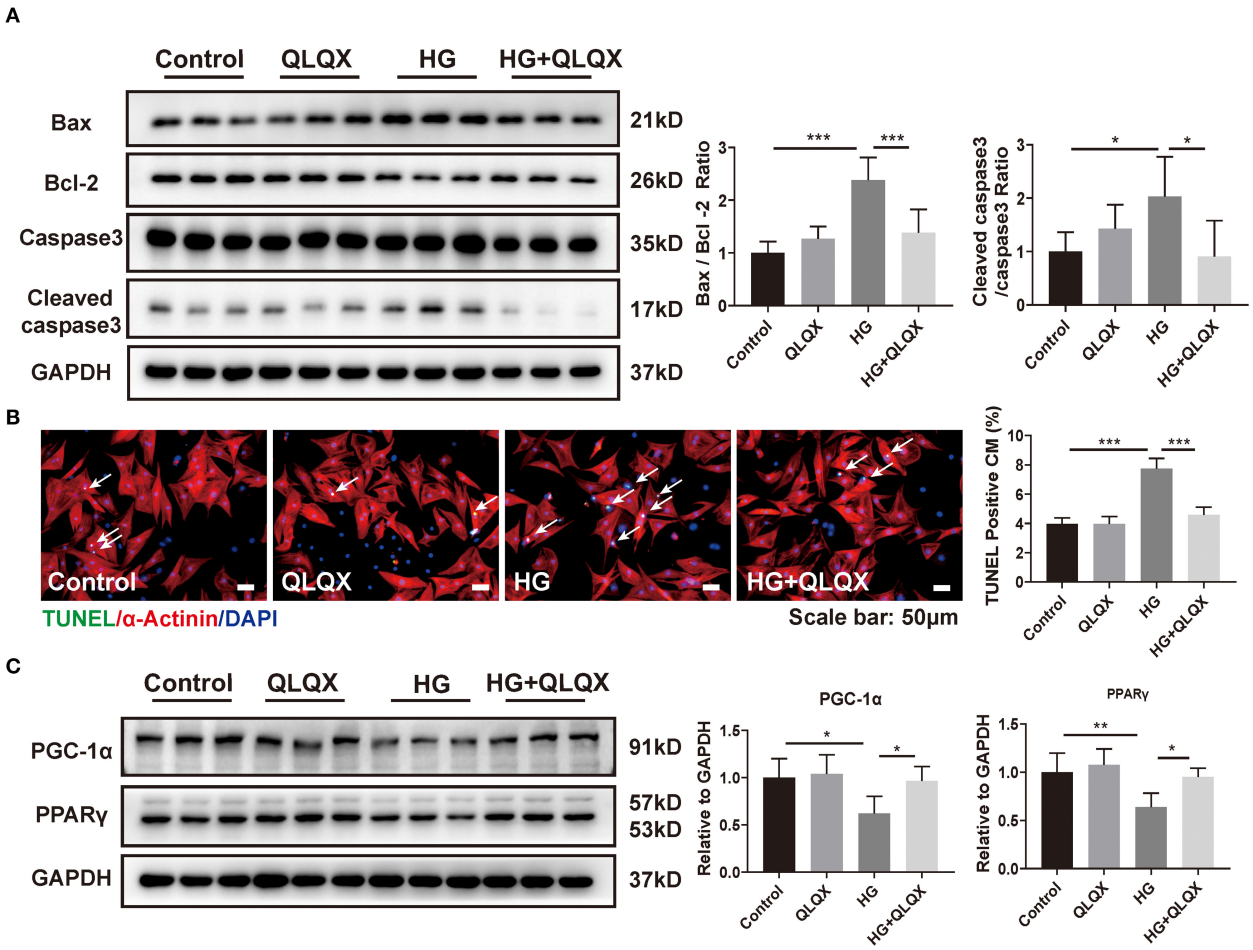
QLQX attenuates HG-induced NRCM apoptosis and activates PPARγ/PGC-1α. **(A,B)** Western blot analysis and TUNEL assay indicated that QLQX attenuated cardiomyocyte apoptosis resulting from HG stimulation. Scale bar: 50 μm (*n* = 6). **(C)** QLQX blocked the reduction effects of HG on expressions of PPARγ and its co-activator PGC-1α (*n* = 6). *p < 0.05; **p < 0.01; ***p < 0.001; vs. respective control.

Our previous studies have demonstrated that QLQX has a beneficial effect on heart failure patients and can attenuate cardiac remodeling via activating PPARγ in post-myocardial infarction mice (16, 17). To investigate whether QLQX could prevent cardiomyocyte from hyperglycemia-induced injury, we incubated NRCMs with QLQX for 48 h prior to the HG exposure and found that QLQX treatment ameliorated HG-induced cardiomyocyte apoptosis (Figures 2A,B). PPARγ is a normal transcriptional regulator which has been reported to be associated with diseases such as diabetes mellitus (25). Then we examined the effect of QLQX on PPARγ/PGC-1α in response to HG treatment. Western blot analysis showed that QLQX treatment blocked the reduction effects of HG on PPARγ and its co-activator PGC-1α expression (Figure 2C). Thus, PPARγ might be the downstream effector of QLQX in alleviating hyperglycemia-induced cardiomyocyte apoptosis.

### QLQX Prevents Hyperglycemia-Induced Apoptosis via Activating PPARγ in NRCMs

**Figure 3:**
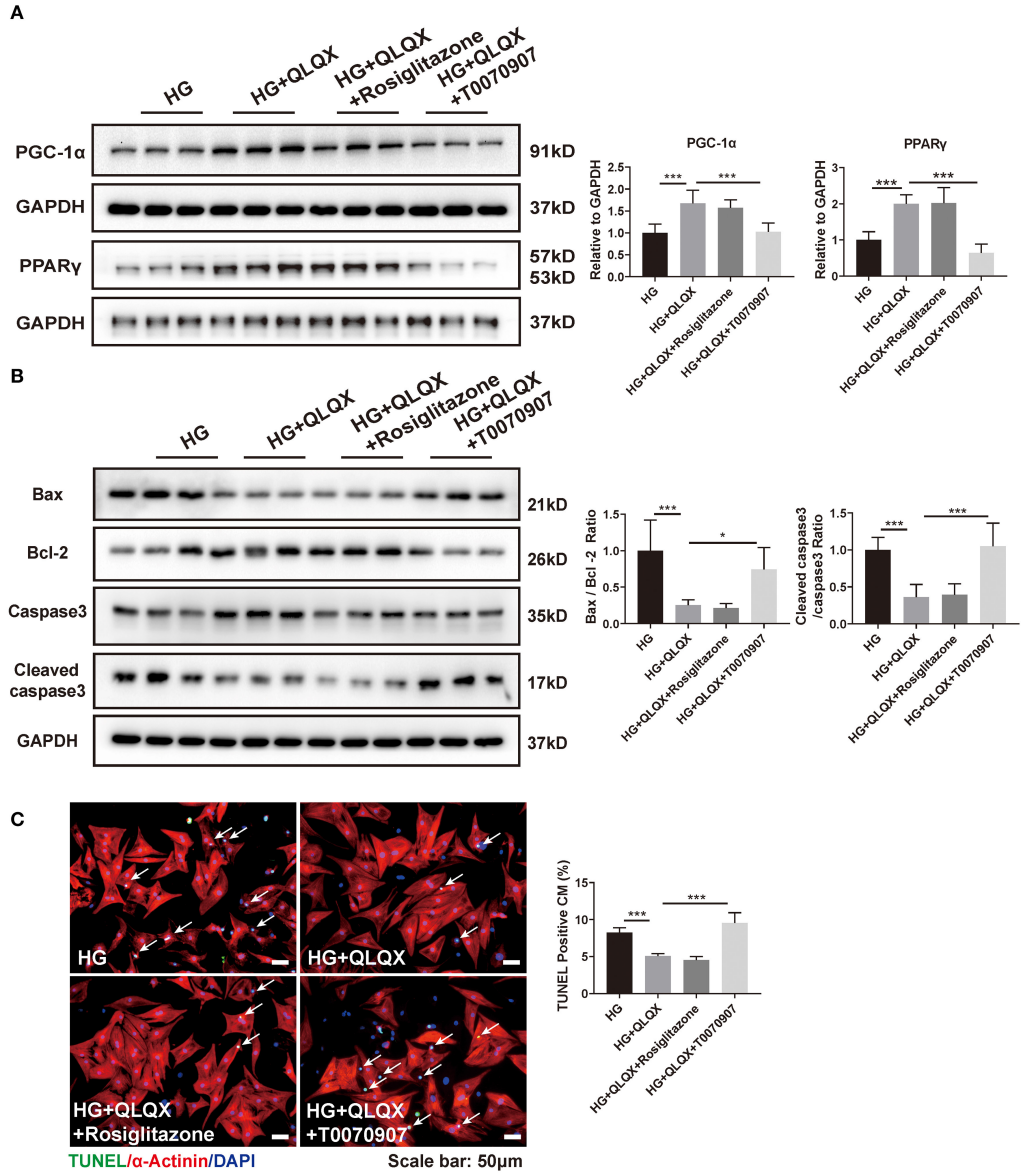
QLQX prevents hyperglycemia-induced apoptosis via activating PPARγ in NRCMs. **(A)** Effect of a PPARγ agonist (rosiglitazone) or inhibitor (T0070907) on QLQX-induced activation of PPARγ and PGC-1α (*n* = 6). **(B,C)** Western blot analysis and TUNEL assay displayed that the PPARγ inhibitor abrogated a cardioprotective effect of QLQX on HG-induced apoptosis. Scale bar: 50 μm (*n* = 6). *p < 0.05; ***p < 0.001; vs. respective control.

To investigate whether PPARγ activation is involved in the regulation of QLQX-mediated cardioprotective effect, a PPARγ agonist (rosiglitazone) and inhibitor (T0070907) were used to stimulate or inhibit the activation of PPARγ. Western blot analysis demonstrated that the PPARγ inhibitor suppressed the expressions of both PPARγ and its coactivator PGC-1α in QLQX-treated cardiomyocytes, but the PPARγ agonist did not exert further increased expression of PPARγ and PGC-1α (Figure 3A). Then, we evaluated the cardiomyocyte apoptosis by western blot and TUNEL staining. As shown in Figures 3B,C, the PPARγ inhibitor blunted the beneficial effect of QLQX in HG-treated cardiomyocytes, while the PPARγ agonist did not demonstrate additional beneficial effects. Collectively, our data suggested that QLQX suppressed cardiomyocyte apoptosis via activating PPARγ.

### QLQX Improved STZ-Induced Cardiac Diastolic Dysfunction and Cardiac Remodeling *in vivo*

**Figure 4:**
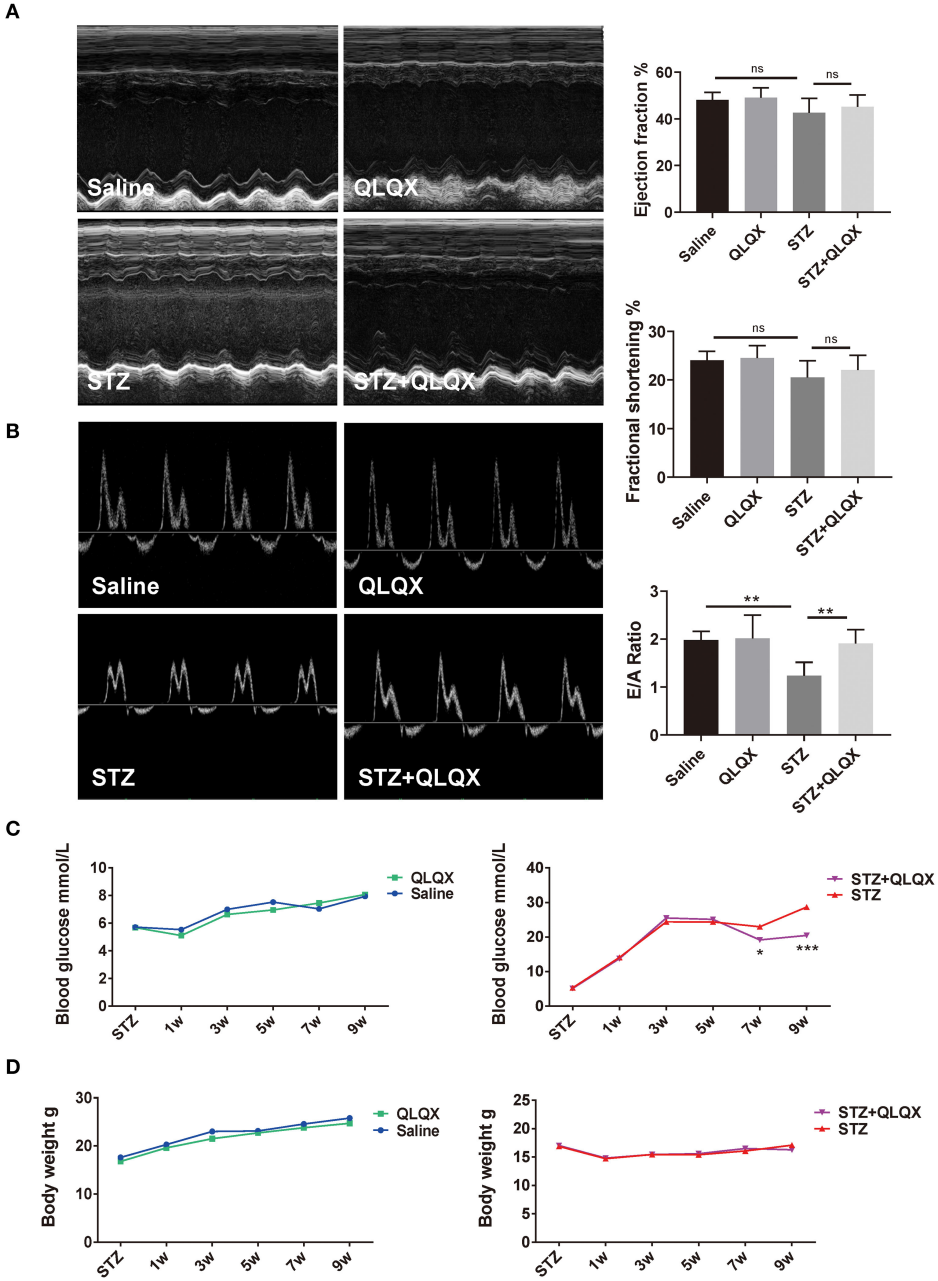
QLQX improved STZ-induced cardiac diastolic dysfunction *in vivo*. **(A)** Echocardiography was used to examine cardiac systolic function of mice, indicated in groups (*n* = 7, 7, 8, and 7). **(B)** Cardiac diastolic function of mice was evaluated using echocardiography, indicated in groups (*n* = 7, 7, 8, and 7). **(C,D)** Blood glucose supervision and body weight were recorded after STZ injection and QLQX treatment (saline group *n* = 9 and QLQX group *n* = 10; at 7 weeks, STZ group *n* = 13 and STZ+QLQX group *n* = 12; at 9 weeks, STZ group *n* = 12 and STZ+QLQX group *n* = 11). *p < 0.05; **p < 0.01; ***p < 0.001; vs. respective control.

To further evaluate the role of QLQX in diabetic cardiomyopathy, we induced diabetes mellitus in mice by STZ injection with QLQX or control treatment. Cardiac function was assessed via echocardiography, as shown in Figures 4A,B, STZ injection induced cardiac diastolic dysfunction with a reduced E/A ratio, while the systolic function such as ejection fraction (EF%) and fractional shortening (FS%) did not display a decline. Compared with the STZ treatment group, QLQX showed a protective effect on diastolic function with an increased E/A ratio, while the systolic function was not altered significantly (Figures 4A,B). Besides, QLQX also displayed a beneficial effect on improving hyperglycemia; the blood glucose level showed a significant reduction after 6 weeks of QLQX treatment (Figure 4C). Moreover, QLQX did not affect the body weight of mice resulting from STZ treatment (Figure 4D).

**Figure 5:**
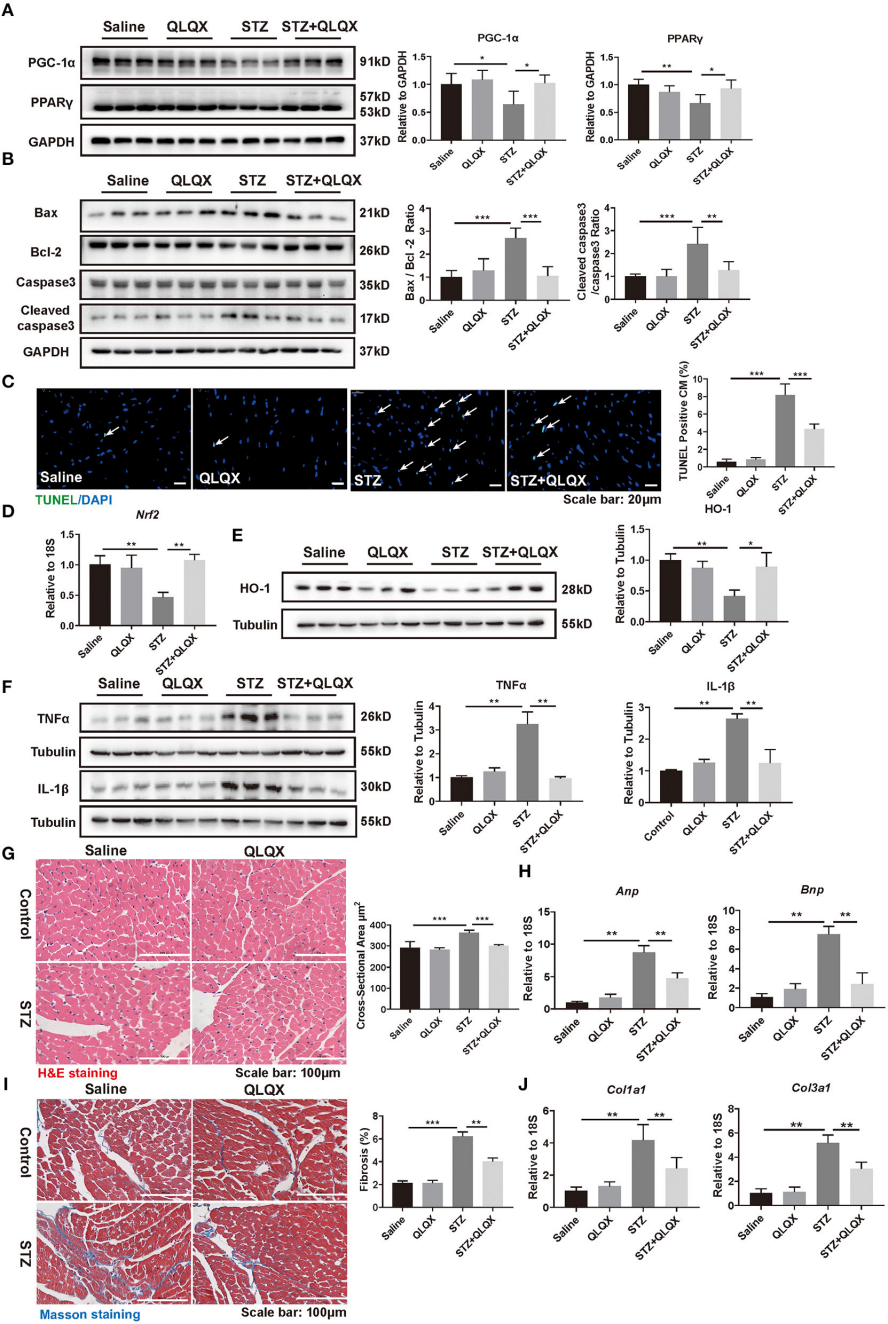
QLQX alleviated STZ-induced cardiac pathological remodeling. **(A)** Western blot analysis of the expression level of PPARγ and PGC-1α in STZ-induced diabetic cardiomyopathy mouse heart tissue, indicated in groups (*n* = 6). **(B,C)** Western blot analysis (*n* = 6) and TUNEL assay (*n* = 6, 6, 6, and 7) suggested that QLQX treatment attenuated cell apoptosis. Scale bar: 20 μm. **(D)** The mRNA level of *Nrf2* was detected using qRT-PCR analysis (*n* = 6). **(E)** Protein level of HO-1 was detected by western blot analysis (*n* = 3). **(F)** Western blot analysis of the expression levels of TNFα and IL-1β, indicated in groups (*n* = 3). **(G)** H&E staining demonstrated the cross-sectional areas of cardiomyocytes, indicated in groups. Scale bar: 100 μm (*n* = 6, 6, 6, and 7). **(H)** qRT-PCR analysis of the expression levels of *Anp* and *Bnp*, indicated in groups (*n* = 6). **(I)** Masson staining was performed to assess cardiac fibrosis, indicated in groups. Scale bar: 100 μm (*n* = 6, 6, 6, and 7). **(J)** qRT-PCR analysis of the expression levels of *Col1a1* and *Col3a1*, indicated in groups (*n* = 6). *p < 0.05; **p < 0.01; ***p < 0.001; vs. respective control.

Consistent with the observation in NRCMs, *in vivo*, QLQX reversed the decreased expressions of PPARγ and PGC-1α which were caused by hyperglycemia-induced diabetic cardiomyopathy (Figure 5A). Also, QLQX treatment attenuated cell apoptosis as determined by western blot (Bax/Bcl-2 and cleaved caspase 3/caspase 3) and TUNEL staining (Figures 5B,C). Besides, QLQX treatment recovered the expression of *Nrf2*, which is a transcription factor that modulated the expression of antioxidant enzymes (Figure 5D). Also, treatment with QLQX increased the expression of HO-1, which is significantly reduced by STZ stimulation (Figure 5E). Furthermore, QLQX suppressed the STZ-treatment-induced expressions of TNFα and IL-1β (Figure 5F).

We further examined the protective effects of QLQX on cardiac structure disturbance. As shown in Figure 5G, H&E staining revealed that enlargement of cardiomyocyte was observed in STZ-treated mice and that the cross-sectional areas of cardiomyocytes decreased in mice treated with QLQX together with STZ. Besides, QLQX blunted the elevation of *Anp* and *Bnp* in STZ-treated mice (Figure 5H). Moreover, cardiac fibrosis was also alleviated by QLQX treatment in mice with STZ-induced diabetic cardiomyopathy as evidenced by Masson staining and the expression levels of *Col1a1* and *Col3a1* (Figures 5I,J). Taken together, the above data demonstrated the beneficial effects of QLQX on diabetic cardiomyopathy.

## Discussion

Chronic diabetes can damage the myocardium (26, 27). Diabetic cardiomyopathy is the primary complication associated with diabetes mellitus and also is the major cause of morbidity and mortality. Limited to specific diagnosis criteria, diabetic cardiomyopathy is often asymptomatic; even some diabetic patients are in great control of glycemia in the original phase (4, 26). Therefore, it is important to prevent or delay the pathological process of diabetic cardiomyopathy. In our present study, we reveal that the traditional Chinese medication QLQX has a cardioprotective role in hyperglycemia-induced cardiomyocyte apoptosis via activating PPARγ *in vitro*. *In vivo*, QLQX treatment attenuated the hyperglycemia-induced cardiac remodeling and improved STZ-induced cardiac diastolic dysfunction.

Uncontrolled hyperglycemia plays a fundamental role in diabetic cardiomyopathy progression and eventually leads to heart failure (28, 29). Sustained hyperglycemia directly induces metabolic alterations in substrate use and mitochondrial oxidation. Usually, cardiac energy substrates contain free fatty acids (FFAs), glucose, ketone bodies, lactate, and amino acids and are mainly dependent on FFAs and glucose oxidation. Under hyperglycemic conditions, FFA oxidation increases, and glucose metabolism reduces. The alterations result in increased lipid uptake, lipid storage, and lipid metabolite, which lead to lipid toxicity and final cardiomyocyte death (7, 10, 11, 15, 30). During the progression of diabetic cardiomyopathy, hyperglycemia leads to increased ROS release and storage and then activates NF-κB signaling and inflammatory response (2, 13, 24). In addition, hyperglycemia could reduce metalloproteinase activity, elevate the accumulation of collagen, lead to myocardial stiffness, and impair contractility (22). Although physical activity has been reported to have beneficial effects on post-meal glucose response and its related cardiovascular diseases, limited pharmacological therapies are available for diabetic cardiomyopathy (31, 32). In this study, we suggest that QLQX could alleviate the cardiac pathogenesis and fibrosis of STZ-induced diabetic cardiomyopathy.

Previous studies have demonstrated that QLQX can attenuate cardiac remodeling after acute myocardial infarction, isoproterenol-induced chronic heart failure, and phenylephrine-induced cardiac hypertrophy (17, 18, 33). Those studies attempted to study the effect of QLQX on pathological cardiac hypertrophy and chronic heart failure. While diabetic cardiomyopathy is often developed in diabetic patients, characterized by metabolic disturbance and myocardial dysfunction (10, 12), the molecular basis of diabetic cardiomyopathy is multifactorial, distinct from acute myocardial infarction and phenylephrine-induced cardiac hypertrophy. Therefore, we raised the hypothesis that QLQX might benefit the diabetic heart; in our present study, we found that QLQX could exert a beneficial effect on diabetics and protect mice from diastolic dysfunction and cardiac remodeling. In this study, by evaluating the effect of QLQX on diabetic cardiomyopathy, we found that QLQX attenuated cardiac remodeling in diabetic hearts. Notably, in diabetic hearts, cardiac dysfunction always progresses from subclinical abnormalities to diastolic dysfunction and eventually to systolic dysfunction (12). Similar to a previous study, our study showed that mice cardiac function was detected after 8 weeks of STZ injection and demonstrated obvious diastolic dysfunction but no difference in systolic function between the saline group and STZ group (4). Some previous studies demonstrated that STZ injection caused both diastolic dysfunction and systolic dysfunction, when the cardiac function is detected at the endpoint of 12 weeks after STZ injection (27, 34). This end-time inconsistency might contribute to the observed difference of diastolic and systolic dysfunction. Collectively, our results support the importance of QLQX in maintaining normal cardiac function.

The transcriptional factor PPARγ is involved in regulating cardiac metabolism (35). PPARγ activation enhances triglyceride synthesis and storage, thus inhibiting lipolysis and modulating fatty acid utilization (35). PPARγ can also co-activate PGC-1α and promote glucose uptake and oxidation (36). Enhanced PGC-1α signaling can subsequently increase NRF2 expression and exert antioxidant effects in hearts (19). Previous studies demonstrated that PPARγ agonists could improve diastolic function and reduce myocardial fibrosis in diabetic hearts (22). QLQX has been reported to improve cardiac energy metabolism and prevent cardiac remodeling in myocardial infarction mice via activating PPARγ (17). Consistently, in this study, the important cardiac metabolism regulator PPARγ contributes to QLQX-mediated diabetic heart benefit; the PPARγ inhibitor treatment would abolish the beneficial effect of QLQX in HG-treated cardiomyocytes.

Cardiomyocyte apoptosis is an important pathophysiological process of diabetic cardiomyopathy (37). Cardiomyocytes undertake continuous elevated blood glucose stimulation which would further facilitate cardiomyocyte apoptosis. Thus, digging effective drugs which can target cardiomyocyte apoptosis would ameliorate the process. In our present study, we suggested that QLQX treatment attenuated cell apoptosis in STZ-induced diabetic cardiomyopathy mice. *In vitro*, the PPARγ inhibitor abolished the beneficial effect of QLQX in HG-treated NRCMs. Taken together, our results demonstrated that QLQX treatment reduced hyperglycemia-induced cardiomyocyte apoptosis and is a promising therapy for diabetic cardiomyopathy. Besides, blood glucose evaluation also causes glycosylation of interstitial proteins and intensifies the formation of ROS, contributing to the impairment of myocardial compliance (10, 12, 38, 39). Interestingly, in this study, we observed that blood glucose level declined after QLQX treatment compared with STZ-only injection. Hyperglycemia reduces the expression of PPARγ (12). The PPARγ agonist has been found to associate with insulin sensitivity and resistance, and the activation of PPARγ could improve cardiac diastolic dysfunction and decrease blood glucose (10, 22). In our study, we found that QLQX could reverse the reduction effects of HG treatment on PPARγ expression. Therefore, the most possible mechanism of QLQX in the blood glucose-reducing effect on diabetic mice might be the activation of PPARγ. However, we cannot exclude the possibility that other factors besides PPARγ might have also contributed to the blood glucose-reducing effect of QLQX. Moreover, though our results indicated a decrease in blood glucose level, it was still over 20 mmol/L in mice, which would further cause cardiac injury. Considering PPARγ plays an important role in cardiac metabolism remodeling and the observed attenuation of cardiac fibrosis, inflammation, and ROS production, we believe that the protective effect of QLQX in the heart might be attributed, but not limited, to the blood glucose reduction effect. Further investigation to elucidate the essential active ingredient and to dig for other regulatory factors of QLQX would provide significant understanding of the QLQX benefit in the heart.

This study has several limitations. First, QLQX contains 11 distinct active components, whether one of the typical herbals in QLQX predominantly acts as the cardioprotective factor or there are cooperative effects among the QLQX is not yet clear. Secondly, though mice received QLQX after the establishment of diabetes *in vivo*, we exposed NRCM to QLQX 48 h pretreatment before HG stimulation *in vitro*. Whether shorter QLQX pretreatment, such as 24 h, or a therapy model such as QLQX treatment after HG stimulation should be applied and whether its underlying mechanism could protect NRCMs from HG-induced apoptosis warrant further investigation.

In our study, we revealed that QLQX inhibited hyperglycemia-induced cardiomyocyte apoptosis in NRCMs via activating PPARγ. We also demonstrated that QLQX attenuated STZ-induced cardiac diastolic dysfunction and cardiac remodeling. Collectively, our data indicated that QLQX represents a potential therapeutic application in the treatment of diabetic cardiomyopathy.

## Data Availability Statement

The raw data supporting the conclusions of this article will be made available by the authors, without undue reservation.

## Ethics Statement

The animal study was reviewed and approved by the Animal Care and Use Committee of Nanjing Medical University.

## Author Contributions

XL, LW, and HS designed the study and provided instructions on all experiments. XW, TZ, PL, MC, GN, HC, and GX performed the experiments and analyzed the data. All authors listed have read and approved this manuscript.

## Conflict of Interest

XL received research grants from Shijiazhuang Yiling Pharmaceutical Co., Ltd. The remaining authors declare that the research was conducted in the absence of any commercial or financial relationships that could be construed as a potential conflict of interest.
