# Supplementary material for: Traditional Chinese Medication Qiliqiangxin Attenuates Diabetic Cardiomyopathy via Activating PPARγ
Source: Front Cardiovasc Med. 2021 Jul 16;8:698056. doi: 10.3389/fcvm.2021.698056 (PMC8322738; doi:10.3389/fcvm.2021.698056)

Supplementary Material

Supplementary Table1: Parameters of echocardiography:

		Control(n=7)	QL(n=7)	STZ(n=8)	STZ+QL(n=7)
**MV E/A Ratio**		1.98±0.18	2.02±0.48	1.24±0.28 **	1.91±0.29 ##
**MV E**	mm/s	615.34±65.65	615.89±74.23	381.28±79.99 **	448.51±109.00
**MV A**	mm/s	313.00±46.48	318.09±67.13	314.77±69.83	241.37±67.50
**Ejection fraction**	%	48.19±3.23	49.12±4.15	42.62±6.19	45.19±5.06
**Fractional shortening**	%	24.01±1.90	24.54±2.53	20.52±3.41	22.06±3.00
**LVPW s**	mm	0.95±0.11	0.81±0.11	0.69±0.15**	0.79±0.13
**LVPW d**	mm	0.70±0.11	0.62±0.08	0.55±0.14	0.58±0.12
**LVID s**	mm	3.15±0.14	3.01±0.20	2.89±0.39	2.89±0.23
**LVID d**	mm	4.15±0.11	3.99±0.19	3.63±0.38**	3.71±0.26
**LV vol s**	µl	39.70±4.16	35.65±5.72	32.73±10.42	32.26±6.16
**LV vol d**	µl	76.50±4.56	69.87±7.89	56.28±13.58**	58.88±9.33

Data are presented as mean ± SD.

**, $p<0.01$, Control group VS STZ group; ##, $p<0.01$, STZ group VS STZ+QL group;

Supplementary Table2: Primers used in qRT-PCR:

Primers	Species	Forward	Reverse
18S	Mouse	GTAACCCGTTGAACCCCATT	CCATCCAATCGGTAGTAGCG
Anp	Mouse	GGAGGAGAAGATGCCGGTAGA	GCTTCCTCAGTCTGCTCACTCA
Bnp	Mouse	AGCTGCTGGAGCTGATAAGAGAA	GTGAGGCCTTGGTCCTTCAA
Col1a1	Mouse	GCTCCTCTTAGGGGCCACT	CCACGTCTCACCATTGGGG
Col3a1	Mouse	CTGTAACATGGAAACTGGGGAAA	CCATAGCTGAACTGAAAACCACC
Nrf2	Mouse	TCTTGGAGTAAGTCGAGAAGTGT	GTTGAAACTGAGCGAAAAAGGC

Supplementary Figure S1. Uncropped scans of Western blots

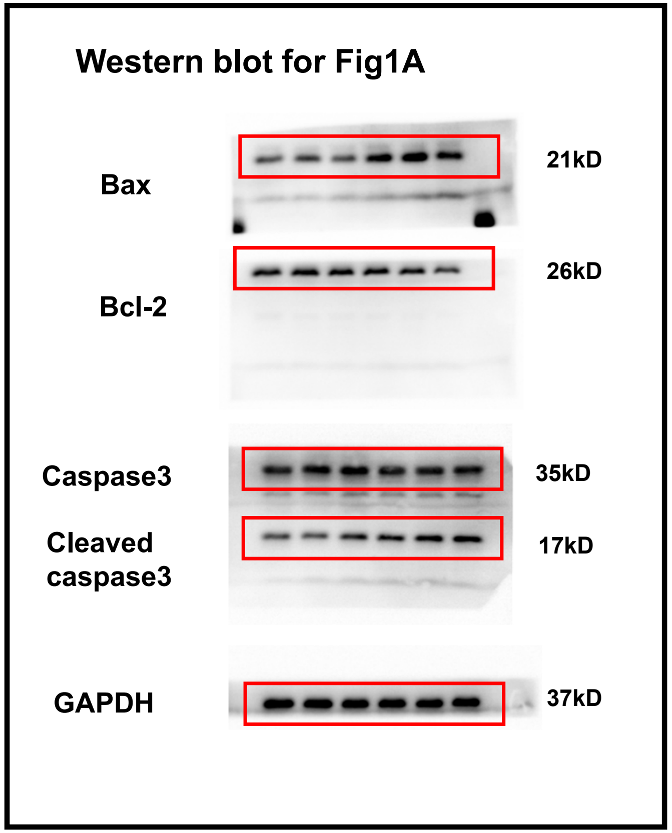

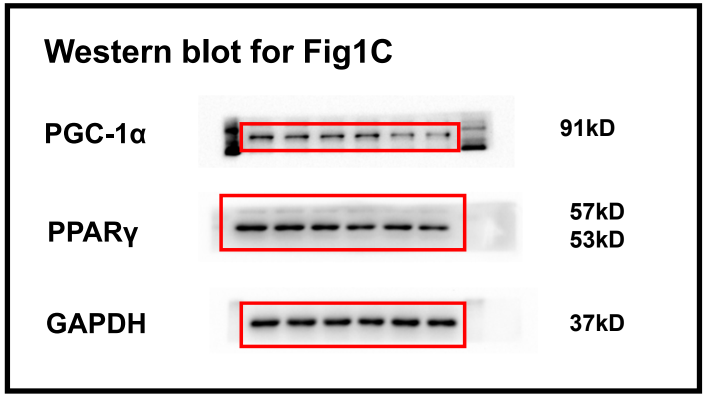

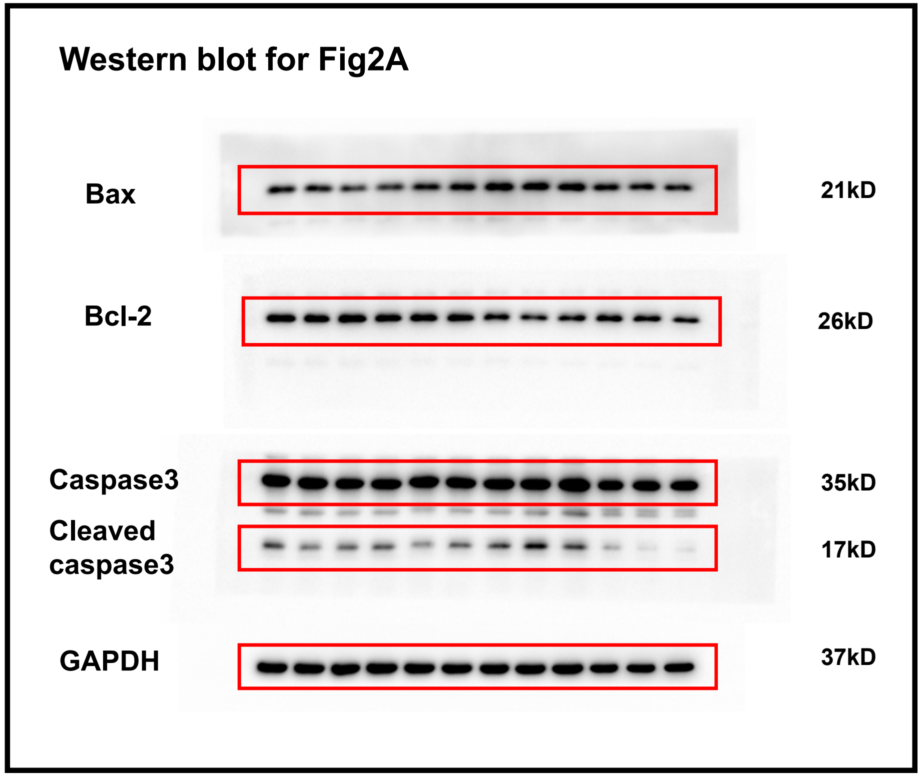
Western blot for Fig2A
Bax
21kD
Bcl-2
26kD
Caspase3
35kD
Cleaved caspase3
17kD
GAPDH
37kD

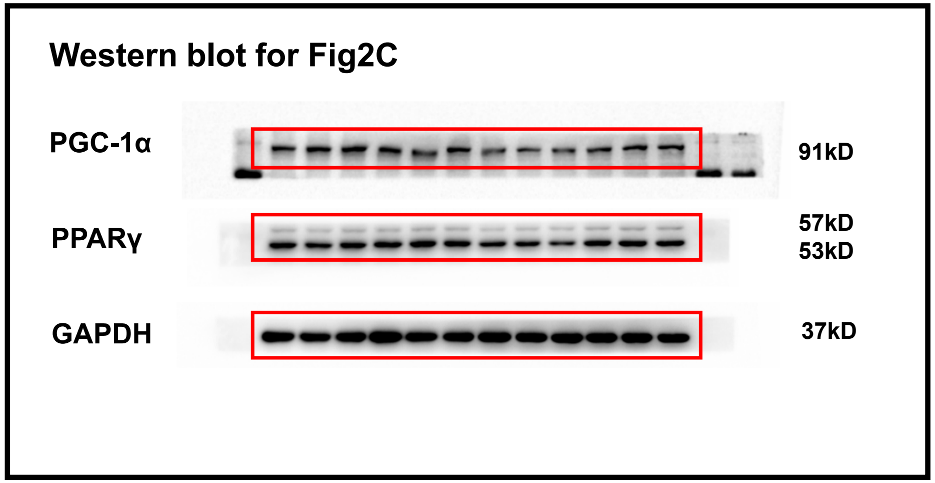
Western blot for Fig2C
PGC-1α
91kD
PPARγ
57kD
53kD
GAPDH
37kD

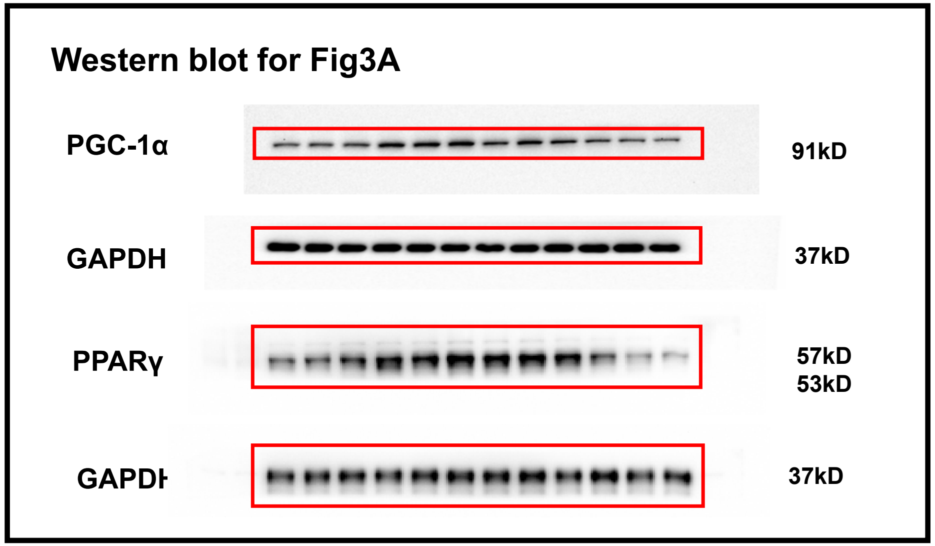
Western blot for Fig3A
PGC-1α
91kD
GAPDH
37kD
PPARγ
57kD
53kD
GAPDH
37kD

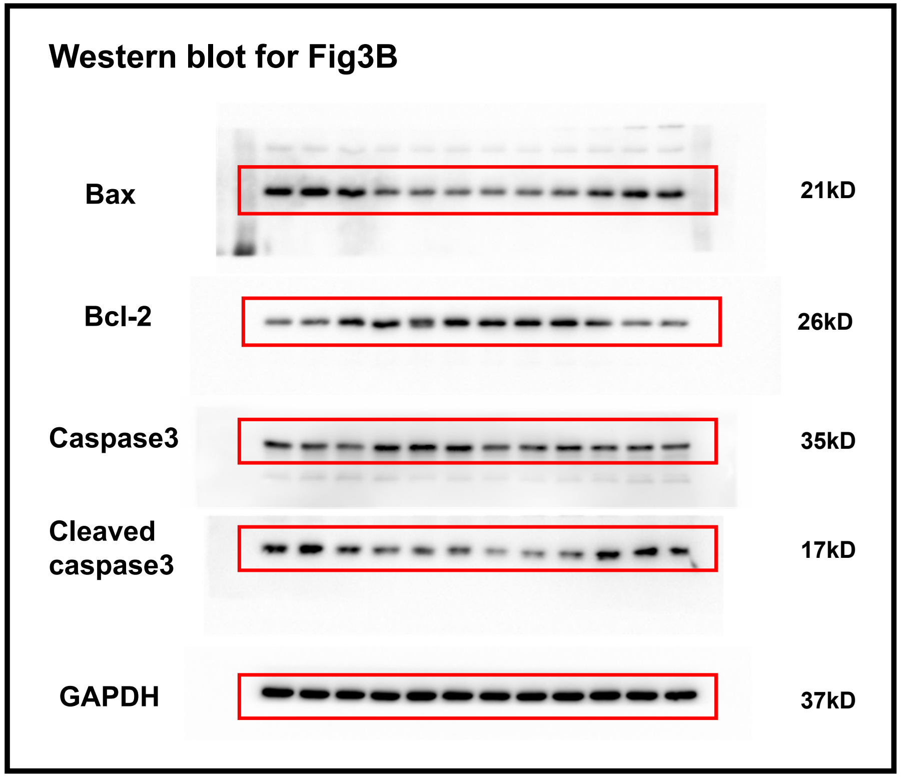
Western blot for Fig3B
Bax
21kD
Bcl-2
26kD
Caspase3
35kD
Cleaved caspase3
17kD
GAPDH
37kD

Western blot for Fig5A

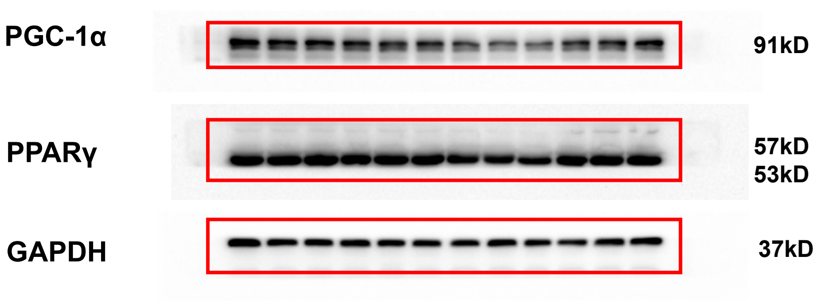

Western blot for Fig5B

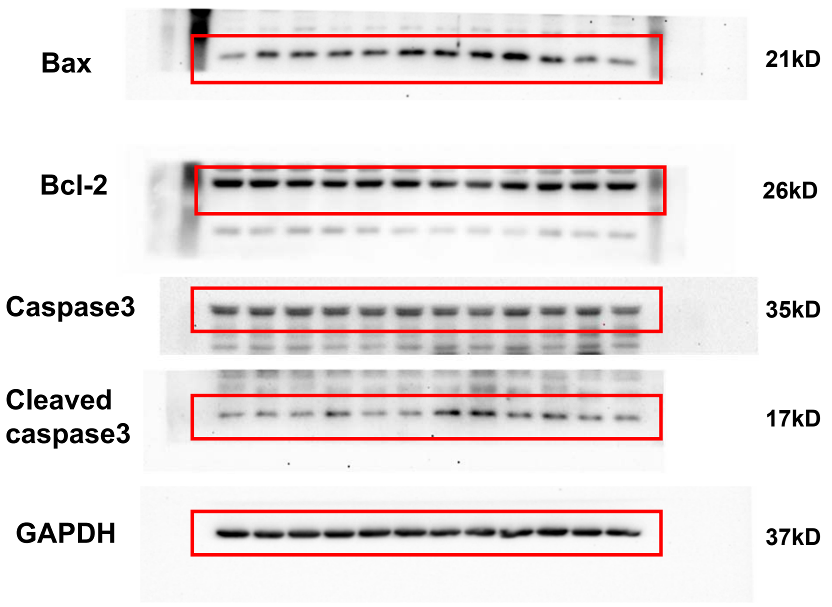

Western blot for Fig5E

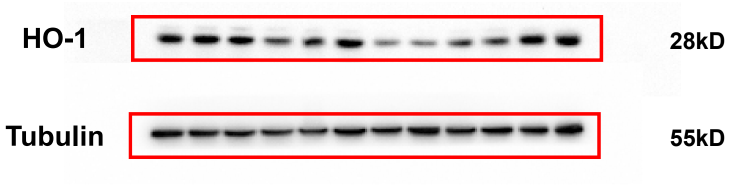

Western blot for Fig5F

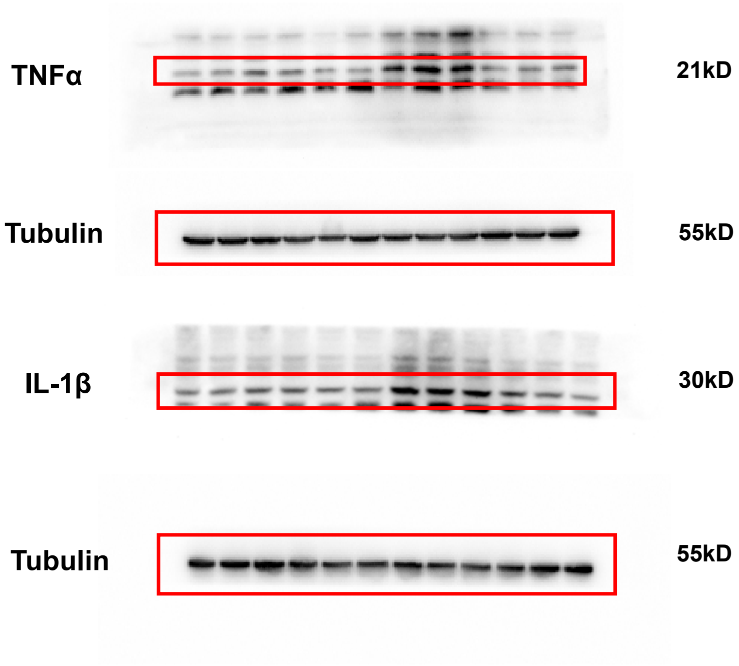

Supplement: Supplementary file 1 [file Data_Sheet_1.pdf]
